# The Combination of CRISPR/Cas9 and iPSC Technologies in the Gene Therapy of Human β-thalassemia in Mice

**DOI:** 10.1038/srep32463

**Published:** 2016-09-01

**Authors:** Zhanhui Ou, Xiaohua Niu, Wenyin He, Yuchang Chen, Bing Song, Yexing Xian, Di Fan, Daolin Tang, Xiaofang Sun

**Affiliations:** 1 Key Laboratory for Major Obstetric Diseases of Guangdong Province, Key Laboratory of Reproduction and Genetics of Guangdong Higher Education Institutes, The Third Affiliated Hospital of Guangzhou Medical University, Guangzhou, Guangdong, 510150, China; 2 Center for DAMP Biology, The Third Affiliated Hospital of Guangzhou Medical University, Guangzhou, Guangdong, 510150, China; 3 Department of Surgery, University of Pittsburgh, Pittsburgh, Pennsylvania, 15213, USA

## Abstract

β-thalassemia results from point mutations or small deletions in the β-globin (*HBB*) gene that ultimately cause anemia. The generation of induced pluripotent stem cells (iPSCs) from the somatic cells of patients in combination with subsequent homologous recombination-based gene correction provides new approaches to cure this disease. CRISPR/Cas9 is a genome editing tool that is creating a buzz in the scientific community for treating human diseases, especially genetic disorders. Here, we reported that correction of β-thalassemia mutations in patient-specific iPSCs using the CRISPR/Cas9 tool promotes hematopoietic differentiation *in vivo*. CRISPR/Cas9-corrected iPSC-derived hematopoietic stem cells (HSCs) were injected into sublethally-irradiated NOD*-scid-IL2Rg−/−* (NSI) mice. HBB expression was observed in these HSCs after hematopoietic differentiation in the NSI mice. Importantly, no tumor was found in the livers, lungs, kidneys, or bone marrow at 10 weeks in the NSI mice after implantation with these HSCs. Collectively, our findings demonstrated that CRISPR/Cas9 successfully corrects β-thalassemia mutations in patient-specific iPSCs. These CRISPR/Cas9-corrected iPSC-derived HSCs express normal HBB in mice without tumorigenic potential, suggesting a safe strategy for personalized treatment of β-thalassemia.

β-thalassemia, one of the most common genetic diseases, is mainly caused by point mutations or small deletions in the beta globin (*HBB*) gene^1^. The condition presents with the total absence or quantitative reduction in globin chain synthesis and ultimately leads to moderate or severe anemia^2^,^3^. The prevalence of carriers of β-thalassemia is 2.54% in southern China and has threatened millions of people’s lives for decades^4^. Despite use of mean corpuscular volume (MCV), mean corpuscular hemoglobin (MCH), and HbA2 values to screen many β-thalassemia carriers, babies with severe anemia are born every year^5^,^6^,^7^. β-thalassemia has two serious types, namely thalassemia major (also termed Cooley’s anemia) and thalassemia intermedia. Patients with these types of β-thalassemia eventually develop a high mortality, resulting in a rising global economical and healthcare burden^8^. At present, hematopoietic stem cell transplantation is the only available definitive cure for patients with the severe forms of β-thalassemia^9^,^10^. However, hematopoietic stem cell transplantation is limited by the paucity of HLA-matched healthy donors for most patients^11^.

Direct reprogramming of somatic cells allows the generation of patient-specific induced pluripotent stem cells (iPSC). Like embryonic stem cells, iPSCs can self-renew, expand on a large scale, and differentiate into all three germ layers (endoderm, mesoderm, and ectoderm), including hematopoietic stem cells^12^,^13^,^14^,^15^. Hematopoietic lineage from pluripotent cells can be achieved by using embryoid body formation in the presence of hematopoietic cytokines or co-culture with stroma cells^16^,^17^.

Recently, the clustered regularly interspaced short palindromic repeats (CRISPR) system has been demonstrated as a powerful tool for targeted genetic modification. The CRISPR-associated 9 (Cas9) nuclease is directed by a 20-nt synthetic single-guide RNA (gRNA) and introduces double-strand breaks into the genome. Compared with other genome editing tools, CRISPR/Cas9 provides powerful tools for enhancing gene-targeting efficiency^18^,^19^ in eukaryotic cells and whole organisms^20^,^21^,^22^,^23^,^24^.

The generation of iPSCs from the somatic cells of β-thalassemia patients with subsequent homologous recombination-based gene correction has raised hopes for curing blood diseases caused by genetic mutations. We and others have shown that the generation of hematopoietic stem cells (HSCs) from the iPSCs of patients with subsequent homologous recombination-based gene correction could recover the production of HBB *in vitro* and improve hemoglobin (HB) production *in vivo*^17^,^25^,^26^,^27^,^28^,^29^,^30^. However, it is unclear whether CRISPR/Cas9-corrected iPSC-derived HSCs *in vitro* perform normal HSC functions *in vivo*.

In this study, we first aimed to determine whether CRISPR/Cas9-corrected β-thalassemia (homozygous 41/42 deletion) iPSCs can differentiate into HSCs and whether those HSCs can survive, differentiate, and produce HBB in NOD*-scid-IL2Rg−/−* (NSI) mice. Secondly, we aimed to study the incidents of tumor formation when CRISPR/Cas9-corrected iPSC-driven HSCs are implanted in NSI mice. We demonstrated that HSCs from CRISPR/Cas9-corrected-iPSCs from β-thalassemia patients can survive, differentiate, and produce HBB in NSI mice without tumorigenic potential. These results greatly support the future clinical application of genetically corrected β-thalassemia patient-specific iPSCs (piPSCs) by CRISPR/Cas9 technologies.

## Results

### Gene-correction of *HBB* mutation in β-thalassemia iPSCs by CRISPR/Cas9

**Figure 1:**
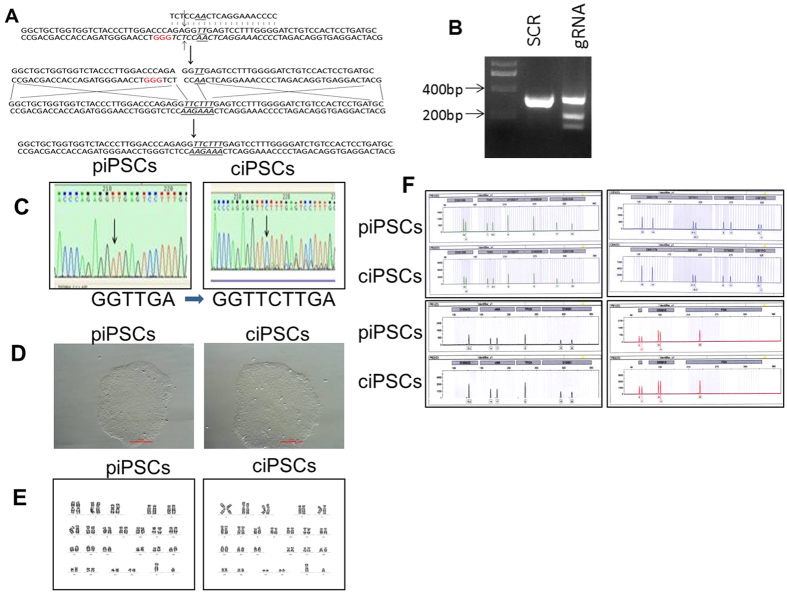
Genetic correction of homozygous 41/42 deletion in piPSCs. (**A**) Schematic representation of the gene-targeting strategy for correcting the homozygous mutation in the *HBB* gene of iPS-41/42 deletion cells using the CRISPR/Cas9 system. (**B**) The cleavage activity of the CRISPR/Cas9 system induced by gRNA using the T7E1 assay. (**C**) Sequencing results of the 41/42 deletion of the *HBB* gene in piPSCs and ciPSCs. The sequence changes are indicated at the bottom. (**D**) Clonal morphology of piPSCs and ciPSCs. (**E**) Karyotype of piPSCs and ciPSCs. (**F**) STR analysis of piPSCs and ciPSCs.

In order to correct the mutation of the *HBB* gene in the piPSCs, CRISPR/Cas9-gRNA was used to cleave the iPSCs genome, whereas the polymerase chain reaction (PCR) products with the normal gene information surrounding the mutation site were used as the donor (Fig. 1A). To confirm Cas9-gRNAs cleavage of the genome of β-iPS-41/42 cells, the Cas9-gRNA vector was transfected into the piPSCs, and then the genomic DNA was extracted following PCR amplification of the surrounding sequence. T7E1 assay confirmed the activity of gRNA (Fig. 1B). To further obtain the gene correction clones, Cas9-gRNA vector plus PCR products were cotransfected into β-iPS-41/42 cells and then selected with puromycin for two days. One week later, the clones were identified by sequencing to observe whether the mutation in the piPSCs was corrected. Indeed, β-thalassemia mutations in piPSCs were corrected by the CRISPR/Cas9 system after sequencing (Fig. 1C). These CRISPR/Cas9-corrected iPSCs (ciPSCs) showed a tightly compacted morphology with high nuclear/cytoplasm ratio clonal (Fig. 1D) and normal karyotypes (46, XY) (Fig. 1E). Short tandem repeat (STR) analyses with 16 loci further revealed the same origin with identical signal peaks in both piPSCs and ciPSCs, suggesting that the ciPSCs were derived from the piPSCs (Fig. 1F). Collectively, these findings indicate that the *HBB* gene mutation in piPSCs is corrected by CRISPR/Cas9.

### Pluripotent stem cell features of ciPSCs

**Figure 2:**
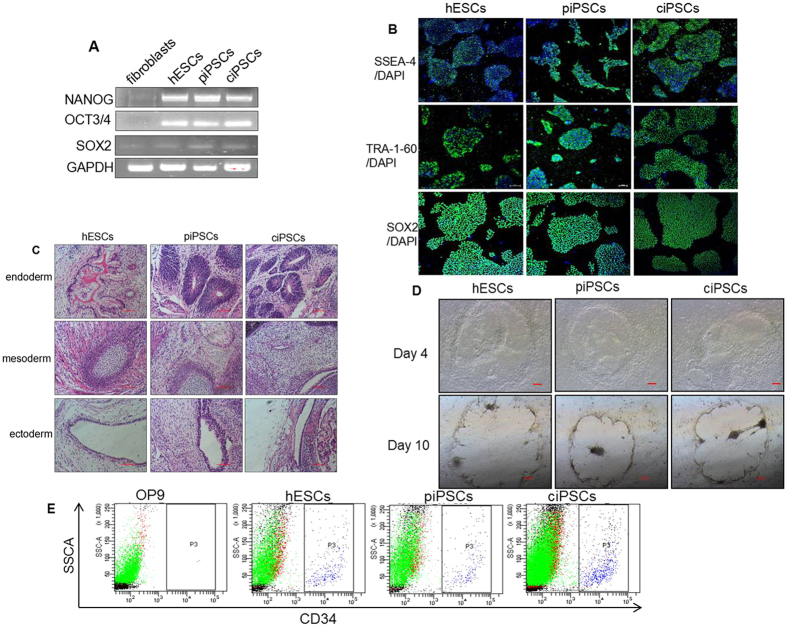
Pluripotent stem cell features of ciPSCs and differentiation *in vivo*. (**A**) RT-PCR analysis of the expression of undifferentiated pluripotent marker genes in ciPSCs. (**B**) Immunostaining of iPS cells for cell surface markers, including SSEA-4, TRA-1-60, and SOX2, scale bar, 20 μm. (**C**) Teratomas that formed eight weeks after injection of iPSCs contained tissues from all three types of germ layers (endoderm, mesoderm, and ectoderm). Scale bars, 100 μm. (**D**) Morphologies of the HSCs of cell lines co-cultured with OP9 stromal cells. (**E**) Flow cytometry analysis results confirm HSC formation *in vitro*.

Octamer-binding transcription factor 3/4 (*OCT3/4*), sex determining region Y-box 2 (*SOX2*), and homeobox rranscription factor nanog (*NANOG*) are widely used markers for pluripotency-associated transcription factors^31^. We therefore assayed whether ciPSCs express these markers by reverse-transcription PCR (RT-PCR) analysis. Indeed, ciPSCs were positive for endogenous expression of *OCT3/4*, *SOX2*, and *NANOG* (Fig. 2A). In addition to pluripotency markers, we also assayed the expression of stem cell markers (e.g., stage-specific embryonic antigen-4 [SSEA-4] and tumor rejection antigen-1-60 [TRA-1-60])^32^ in ciPSCs by immunofluorescence analysis. We found that these ciPSCs stain positive for SSEA-4, TRA-1-60, as well as SOX2 (Fig. 2B). These findings demonstrated pluripotent stem cell features of ciPSCs *in vitro*.

To further confirm these findings *in vivo*, cells were subcutaneously injected into immunodeficient (SCID) mice. A significant change to teratoma formation in SCID mice was observed at eight weeks after injection of ciPSCs. Histological examinations further confirmed that the teratomas contained various tissues comprising all three germ layers (endoderm, mesoderm, and ectoderm) (Fig. 2C). As a positive control, human embryonic stem cells (hESCs) exhibited a similar phenotype as ciPSCs in pluripotency experiments. There were no differences between these iPSCs and hESCs in their ability to either self-renew or differentiate.

### Hematopoietic differentiation *in vitro*

Stem cell co-culture with stromal cell lines such as S17, OP9, and fetal liver-derived stromal cells can result in spontaneous generation of hematopoietic cells^33^. To assay the hematopoietic differentiation ability of piPSCs and ciPSCs, all iPSCs and hESCs were co-cultured with OP9 stromal cells, separately. These cells exhibited dynamic similarity in morphology (Fig. 2D). Notably, both uncorrected and corrected ciPSCs had similar abilities to differentiate and produce HSCs after co-culture with stromal cells. Flow cytometry analysis showed that the proportion of CD34 + cells derived from the piPSCs and ciPSCs at day 10 was 2.8% and 3.1%, respectively (Fig. 2E). These results demonstrated that both piPSCs and ciPSCs undergo successful hematopoietic differentiation *in vitro*.

### Hematopoietic differentiation *in vivo*

NSI mice lack mature T cells, B cells, and natural killer cells and therefore permit the engraftment of a wide range of primary human cells. We therefore used NSI mice to observe hematopoietic differentiation of ciPSCs *in vivo*. Female NSI mice with similar weights were sub-lethally irradiated with 1.0 Gy/min X-ray for 1 min and then randomly divided into six groups: the α-MEM group, piPSC group, ciPSC group, niPSC (iPSCs derived from normal human blood) group, UCB (Umbilical Cord Blood) group, and the hESC group. Then, 5 × 10^5^ CD34 + HSCs derived from these cells were enriched by magnetic-activated cell sorting (MACS) and transplanted into the right femurs of the irradiated NSI mice. Routines blood tests were performed every week for four weeks after transplantation. Both HB and red blood cells (RBCs) in the peripheral blood dropped after irradiation (P < 0.05). HB levels in the ciPSC group, niPSC group, UCB group, and hESC group were higher than in the α-MEM group and piPSC group at two weeks after transplantation (Table 1). However, there were no differences among these groups for all weeks and indexes (P > 0.05).

**Figure 3:**
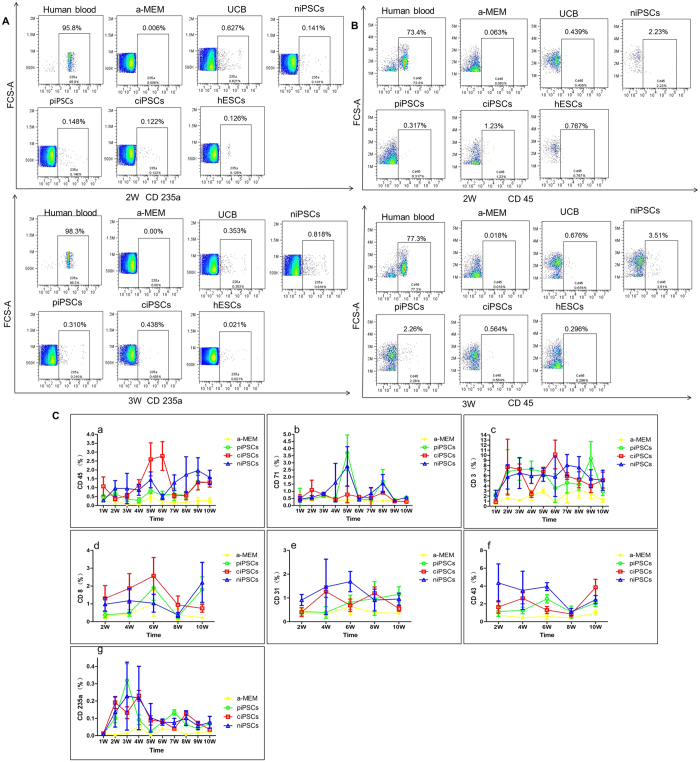
Flow cytometry analyses of anti-human CD3, CD45, CD71, CD235a, CD8, CD31, and CD43 after transplantation in the blood. (**A**) Flow cytometry results of anti-human CD45 and CD235a in the second and third weeks. (**B**) Flow cytometry analyses of anti-human CD3, CD45, CD71, CD235a, CD8, CD31, and CD43 every week or every other week after transplantation. (**C**) Consecutive test results of the α-MEM, piPSC, ciPSC, and niPSC groups after transplantation.

The cluster of differentiation (often abbreviated as CD) is widely used for the identification and immunophenotyping of cells. We next analyzed CD marks in the blood using flow cytometry analysis. Anti-human CD3 (present on 70–80% of normal human peripheral blood lymphocytes and 60–85% of thymocytes), anti-human CD45 (present on all human leukocytes including lymphocytes, monocytes, granulocytes, eosinophils, and thymocytes), anti-human CD71 (expressed on erythroid progenitors), and anti-human CD235a (expressed on human erythrocytes and erythroid precursor cells) were performed every week, whereas anti-human CD8 (expressed by the majority of thymocytes), anti-human CD31 (expressed on platelets, monocytes, granulocytes, and in high amounts on endothelial cells), and anti-human CD43 (expressed on T cells, pre-B cells and activated B cells, NK cells and granulocytes) were detected every other week after transplantation. The levels of these CD markers in the piPSC, ciPSC, niPSC, UCB, and hESC groups were higher than those in the α-MEM group (Fig. 3A,B). Analysis of the α-MEM, piPSC, ciPSC, and niPSC groups are also shown in Fig. 3C.

**Figure 4:**
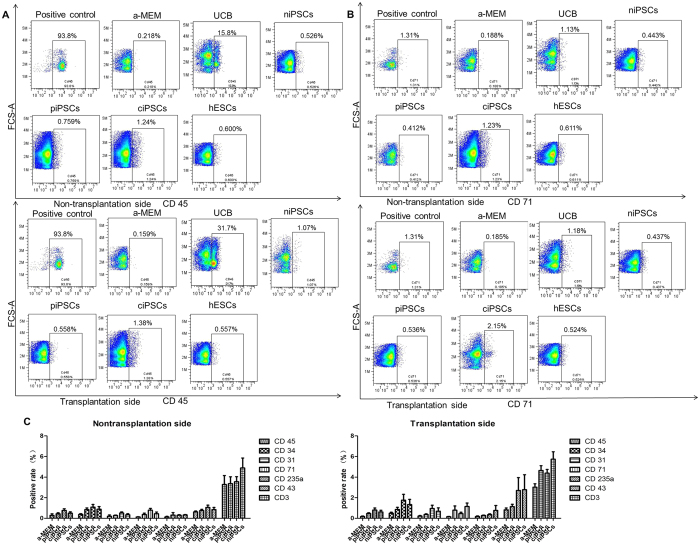
Flow cytometry analyses of anti-human CD3, CD45, CD71, CD235a, CD34, CD31, and CD43 in the blood marrow (BM). (**A**) Flow cytometry results of anti-human CD45 in the BM of all groups. (**B**) Flow cytometry results of anti-human CD71 in the BM of all groups. (**C**) Analysis of BM in the α-MEM, piPSC, ciPSC, and niPSC groups.

All of the mice were sacrificed and the bone marrow (BM) cells from the transplantation (right femur) and the non-transplantation sides (left femur) were collected and analyzed with flow cytometry using specific anti-human CD3, CD45, CD71, CD235a, CD34 (expressed on hematopoietic progenitor cells), CD31, and CD43 at 10 weeks after transplantation. The levels of these CD markers in the piPSC, ciPSC, niPSC, UCB, and hESC groups were higher than those in the α-MEM group. Analysis of the α-MEM, piPSC, ciPSC, and niPSC groups is shown in Fig. 4. Analyses of these CD markers in the peripheral blood and BM demonstrated that hematopoiesis of the transplanted human cells occurred in the NSI mice.

**Figure 5:**
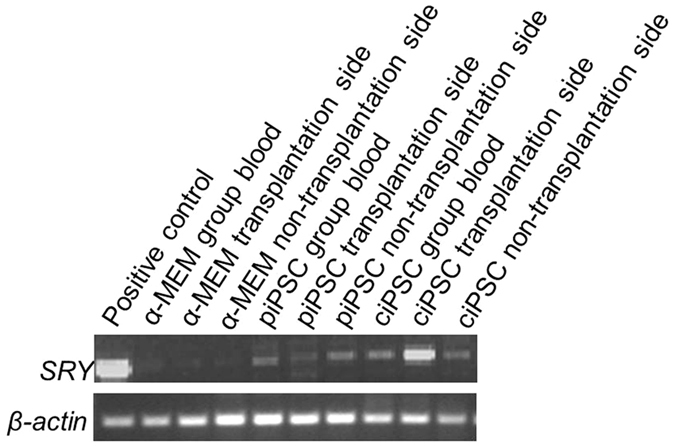
Human *SRY* gene detected in the blood and BM cells from the transplantation and the non-transplantation sides of the female NSI mice.

The sex-determining region Y (*SRY*) is a gene found on Y chromosomes that leads to the development of male phenotypes. RT-PCR was further used to detect the human *SRY* gene in the blood and BM cells from the transplantation and the non-transplantation sides of the female NSI mice. If the ciPSC transplant fails, no human *SRY* gene can be tested in female NSI mice. The human *SRY* gene was detected in the ciPSC group (Fig. 5), further confirming that hematopoiesis of the transplanted human cells had occurred in the NSI mice.

**Figure 6:**
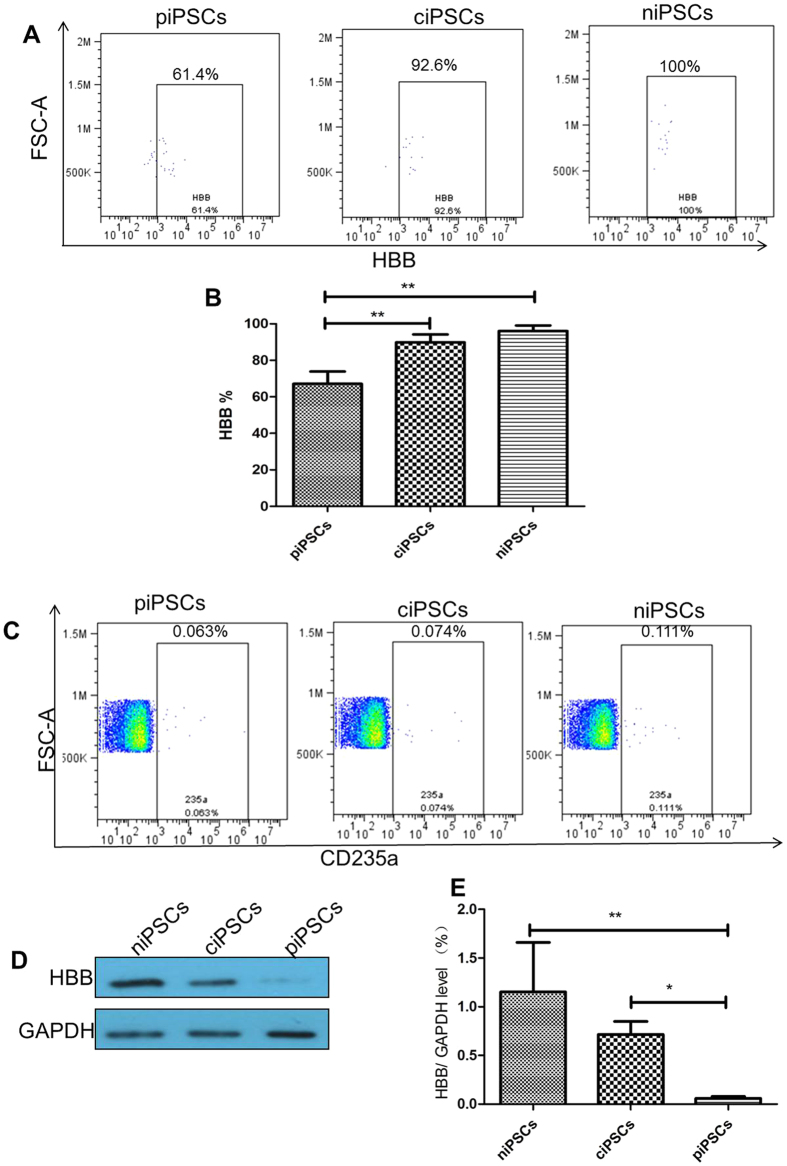
HBB proteins were detected with flow cytometry and western blot analysis 10 weeks after transplantation. (**A**) Flow cytometry analysis with specific anti-human HBB in the anti-human CD235a positive cells. (**B**) Flow cytometry analysis of the HBB levels. **P < 0.01, n = 5. (**C**) Flow cytometry sorted with specific anti-human CD235a. (D) CD235a cells were used to detect HBB proteins with western blot. (**E**) The results of HBB protein levels in the ciPSC, piPSC, and niPSC groups. *P < 0.05, **P < 0.01, n = 3.

To address whether ciPSC-derived HSCs can produce HBB proteins *in vivo*, we further tested HBB proteins levels using two different testing methods. First, 2 μl of mouse peripheral blood cells underwent flow cytometry analysis with specific anti-human CD235a and anti-human HBB. Indeed, CD235a and HBB positive cells were found in both the piPSC and ciPSC groups, but the ratio of HBB positive cells in the ciPSC group was higher than that in the piPSC group (P < 0.01) (Fig. 6A,B). Secondly, 1 ml of peripheral blood cells of the mice was sorted with specific anti-human CD235a by flow cytometry. These CD235a positive cells were further used to detect HBB protein levels by western blot analysis. The expression of HBB protein in the ciPSC group was higher than that in the piPSC group (P < 0.05) (Fig. 6C–E). These findings indicate that ciPSC-derived HSCs can produce normal HBB proteins *in vivo*.

### Pathological results

**Figure 7:**
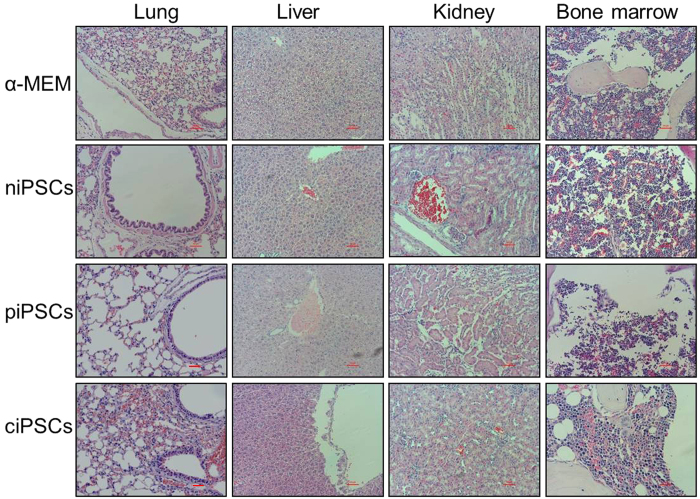
Pathological results of mouse livers, lungs, kidneys, and bone marrow. Scale bars, 100 μm.

The potential tumorigenicity of iPSCs is a major concern in the clinical development of human iPSCs for therapy. Key factors that influence the tumorigenicity of iPSCs include the reprogramming method, the selection of clonal lines, and how the iPSCs are treated thereafter. We next evaluated the tumorigenic potential of ciPSCs in tissues from mice by histopathology analysis. Indeed, no tumors were observed in the livers, lungs, kidneys, or BM 10 weeks after injection (Fig. 7). Moreover, no unexplained premature deaths were observed after these mice received transplants. Thus, our studies indicate that ciPSC-derived HSCs express normal HBB in mice without tumorigenic potential.

## Discussion

Advances in the development of genome engineering tools are allowing us to cure human diseases caused by genetic mutations. β-thalassemia syndromes are a group of hereditary disorders characterized by a genetic deficiency in the synthesis of HBB. We and others have shown that the generation of HSCs from the iPSCs of patients with subsequent gene correction could recover the production of HBB^17^,^25^,^26^,^27^,^28^,^29^. Notably, the CRISPR/Cas9 system is the most popular homologous recombination-based gene correction method, which has proven to be a powerful tool for targeted genetic modification^17^,^29^,^34^,^35^. In our study, the genetically corrected homozygous 41/42 deletion of patient-specific iPSCs (one allele of the mutated *HBB* gene corrected) also maintained normal karyotypes (46, XY), retained their pluripotency, and differentiated into HSCs. However, before the clinical application of iPSCs, a number of hurdles must be overcome. Mysteries remain, such as the ability of HSCs from the gene correction of patient iPSCs *in vivo* to survive and differentiate; HSC recovery of the production of HBB *in vivo*; and whether tumors form after transferring HSCs. Therefore, we focused on HSCs differentiated from CRISPR/Cas9-corrected iPSC *in vivo*.

In this study, we used NSI mice, which with a more severely impaired immune system, attained a higher tumor engraftment index score and were more suitable for xenograft and allograft experiments^36^. Typical routine blood test results showed that the levels of both HB and RBCs in the peripheral blood dropped after irradiation. HB levels of transplanted mice in the ciPSC, niPSC, UCB, and hESC groups were more highly elevated than those in the α-MEM group and piPSC group at two weeks after transplantation, but there was no statistical significance. Unlike our study, another study indicated that genetically corrected iPSC-derived HSCs could positively stimulate hematopoiesis in SCID mice after irradiation^26^. Some of the possible reasons for the difference would be the change in numbers, strains of animals, and quality of the HSCs.

In order to confirm that HSCs from the iPSCs of patients could survive and differentiate *in vivo*, flow cytometry analysis of human CD markers were performed. Anti-human CD3, CD45, CD71, CD31, CD34, CD43, and CD235a were detected in BM cells from the transplantation and the non-transplantation sides of the mice. The presence of the human *SRY* gene was also detected in genomic DNA extracted from the peripheral blood and BM cells from both sides of the cell-transplanted female NSI mice. These findings confirmed that the transplanted human cells underwent hematopoiesis in the NSI mice.

Furthermore, flow cytometry analysis and western blot were used to detect human HBB proteins in the peripheral blood. First, flow cytometry analysis with specific anti-human CD235a and anti-human HBB was performed. Secondly, flow cytometry was sorted with specific anti-human CD235a, and then the CD235a cells were used to detect the HBB proteins with western blot. Both analyses suggested that the genetically corrected homozygous mutation of β-thalassemia iPSCs could produce HBB after hematopoietic differentiation *in vivo*. These results were consistent with findings from a previous study using quantitative PCR analysis, high-performance liquid chromatography, and western blot analysis^26^.

In summary, we demonstrated that HSCs from the correction of homozygous mutation of β-thalassemia iPSCs could survive, differentiate into RBCs, and produce β-globin after hematopoietic differentiation *in vivo*. There were no observations of tumor formation in NSI mice after ciPSC transplantation. Our results highlight a safe gene therapy strategy of combining iPSCs and CRISPR/Cas9 technology to treat β-thalassemia. Further studies are needed to observe the innate immune response to this bacterial original CRISPR/Cas9 genome editing tool.

## Methods

### Ethics statement

All experimental protocols were approved by The Third Affiliated Hospital of Guangzhou Medical University. All animal experiments were approved by the Animal Ethics Committee of The Third Affiliated Hospital of Guangzhou Medical University and performed in accordance with the guidelines of Animal Care and Use of The Third Affiliated Hospital of Guangzhou Medical University. All participants gave written informed consent to participate in the study. Ethical approval was given by the Ethical Committee of The Third Affiliated Hospital of Guangzhou Medical University and all the methods were carried out in accordance with the approved guidelines.

### Feeder-free culture of hESCs and iPSCs

Human embryonic stem cells (hESCs)^37^, niPSCs (iPSCs derived from normal human blood), piPSCs (the β-iPS-41/42 cell line was generated using cells from a β-thalassemia patient with a homozygous CD41/42 (-CTTT) *HBB* mutation and the Sendai virus)^30^, and ciPSCs (β-thalassemia patient-specific iPSCs with genetically corrected homozygous 41/42 deletion) were established in the Key Laboratory for Major Obstetric Diseases of Guangdong Province, The Third Affiliated Hospital of Guangzhou Medical University. Cell lines were cultured using Essential 8 Medium (Gibco, USA)/Geltrex LDEV-Free hESC-qualified Reduced Growth Factor Basement Membrane Matrix (Invitrogen, USA) in a feeder-independent culture system according to the instructions from the manufacturer.

### gRNA assembly and PCR donor purification

Sequences used for cloning the gRNA into the PX459 vector were 5′-CACCGCCCCAAAGGACTCAACCTC-3′ and 5′-AAACTTGGACCCAGAGGTTGAGTCC-3′. To prepare the PCR donor, we used the primers 5′-GAGAAGACTCTTGGGTTTCTGATAG-3′ and 5′-CAGCTCACTCAGTGTGGCA-3′ to amplify, and used the PCR products as donor. 5′-GACAGAGAAGACTCTTGGGTTTCTGATA-3′ and 5′-CAGCTCACTCAGTGTGGC A-3′ were used to perform the T7E1 assay.

### RT-PCR

Total RNA was isolated from cells using a TRIzol kit (Invitrogen, USA). A PrimeScript RT Reagent Kit (TaKaRa, Japan) was used to synthesize cDNA from 1 μg of RNA. RT-PCR was performed to detect the expression of endogenous pluripotency genes. The primers were used as follows: *Oct3/4*, 5′-GACAGGGGGAGGGGAGGAGCTAGG-3′ and 5′-CTTCCCTCCAACCAGTTGCCCCAAAC-3′; *Nanog*, 5′-CAGCCCCGATTCTTCCACCAGTCC-3′ and 5′-CGGAAGATTCCCAGTCGGGTTCACC-3′; *SOX2*, 5′-ACCAGCTCGCAGACCTACAT-3′ and 5′-ACTTGACCACCGAACCCAT-3; *GAPDH* (glyceraldehyde-3-phosphate dehydrogenase), 5′-CCTTCATTGACCTCCACTAC-3′ and 5′-GTTGTCATACTTCTCATGGTT-3′. RT-PCR was also used to detect the human *SRY* gene, *β-actin,* 5′-GATGGTGGGAATGGGTCAGA-3′ and 5′- CCTATGGGAGAACGGCAGA-3′ *SRY,* 5′-TGAAGCGACCCATGAACG-3′ and 5′- GATCTGCGGGAAGCAAACT-3′.

### Karyotype analysis

For chromosome analysis, iPSCs were incubated in culture medium with 0.25 g/mL colcemid (Gibco, USA) for 3 h, harvested, incubated in 0.4% sodium citrate and 0.4% chloratum: kaliumat (1:1, v/v) at 37 °C for 5 min, and then fixed in methanol:acetic acid (3:1, v/v) three times. After Giemsa staining, at least 20 cells were examined in each group for chromosome analysis.

### STR analysis and DNA sequencing analysis

For STR analysis, genomic DNA was extracted from piPSCs and ciPSCs, respectively, and used as templates for PCR. STR signal analysis was performed according to the Promega PowerPlex 16 System instructions (Promega, USA). For sequencing analysis, the mutation sites of *HBB* genes were amplified by PCR from genomic DNA of piPSCs and ciPSCs and sequenced by BGI Life Tech Company. The sequences of *HBB* sequencing primers were: 5′-CACTAGCAACCTCAAACAGACA-3′; 5′-CTCAAGGCCCTTCATAATATCC-3′.

### Immunofluorescent staining

Cells were fixed in 4.0% paraformaldehyde for 20 min, permeabilized with 0.5% Tween-20 for 30 min, incubated with primary antibody overnight, and incubated with secondary antibodies (Alexa Fluor; Invitrogen) for 1 h. The cells were imaged using an inverted confocal microscope (Nikon, Japan). The primary antibodies used in this study were as follows: anti-SSEA-4 (1:100; Sigma, St. Louis, MO, USA), anti-TRA1-60 (1:200; Sigma), and anti-SOX2 (1:200; Sigma). For nuclear staining, DAPI (4, 6-diamidino-2-phenylindole; Sigma) was used.

### Teratoma formation

The iPSCs were harvested by mechanical dissociation and collected into tubes with 200 μL DMEM/F12 (Invitrogen). Cells from a 60-mm dish were injected subcutaneously into the groin of a SCID mouse. Eight weeks after injection, the tumors were dissected and fixed with phosphate buffered saline (PBS) containing 10% paraformaldehyde. Paraffin-embedded tissue was sliced and stained with hematoxylin and eosin.

### Hematopoietic differentiation

For cell differentiation, OP9 cells were plated onto gelatinized 100-mm dishes in α-MEM (Invitrogen) containing 10% fetal bovine serum (FBS) (BI), 2 mM L-glutamax (Invitrogen), and 50 μg/ml penicillin/streptomycin (Gibco). After formation of confluent cultures on day 4, half of the medium was changed and cells were cultured for an additional two to three days. Undifferentiated hESCs and iPSCs were harvested by mechanical dissociation into small clumps and added to OP9 cultures at a density of 1.5 × 10^6^/20 ml per 100-mm dish in α-MEM supplemented with 10% FBS, 100 μM monothioglycerol (MTG; Sigma), 100 μM vitamin C (Sigma), 10 ng/ml IL-3 (Peprotech), 50 ng/ml SCF (Peprotech), 50 ng/ml IL-6 (Peprotech). Co-cultures were incubated for up to 10 days with a half-medium change every other day. Cells were harvested on day 10, and single-cell suspension was prepared for clonogenic, flow-cytometric assays, and MACS for CD34 + cells.

### MACS cell sorting for CD34 + cells

Cells from hESCs/OP9, iPSCs/OP9 co-cultures, and human umbilical cord blood were collected, washed with MACS buffer (PBS with 2% FBS), and incubated with CD34 MicroBeads (Miltenyi Biotec) for magnetic labeling. Then cells were loaded onto the column. Magnetic separation was performed in the magnetic field according to instructions.

### Bone marrow transplantation

Twelve to 24 hours before transplantation, NSI (NOD*-scid-IL2Rg−/−,* provided from Dr. Peng Li, which with a more severely impaired immune system, attained a higher tumor engraftment index score and were more suitable for xenograft and allograft experiments^36^) mice at the age of six to eight weeks old were sublethally irradiated with 1.0 Gy/min for 1 min (RS2000 X-ray). Mice were randomly divided into six groups: the α-MEM group (n = 4), piPSC group (n = 5), ciPSC group (n = 5), niPSC group (n = 4), UCB group (n = 3), and the hESC group (n = 4). For transplantation, 5 × 10^5^ MACS sorted CD34 + HSCs were collected and resuspended in 20 μl α-MEM medium. After anesthesia, the cell suspension in the 28 G insulin syringe was gently injected into the right femurs of the mice.

### Routine blood tests

Routine tests were performed on 30 μl of mouse blood following the manufacturer’s instructions (Sysmex XN-10) for dilution mode.

### Pathological examination

All of the mice were sacrificed after 10 weeks and pathological examinations of livers, lungs, kidneys, and BM were performed.

### Flow cytometry assay and cells sorting

For analysis of HSC differentiation efficiency, hESCs/OP9 and iPSCs/OP9 co-cultures were collected and washed with FACS buffer (PBS with 2% FBS). Cells were stained with anti-human CD34-PE-Cy7 monoclonal antibody (BD). For analysis of human cell ratio in the mice, the peripheral blood cells were collected and BM cells were flushed from the femurs of mice. After washing with FACS buffer and red cell lysis (Ebioscience), cells were stained with anti-human CD235-APC monoclonal antibody (BD) (no need for red cells lysis) and anti-human CD3-FITC monoclonal antibody (BD), anti-human CD8-PE-Cy7 monoclonal antibody (BD), anti-human CD31-PE monoclonal antibody (BD), anti-human CD34-PE-Cy7 monoclonal antibody (BD), anti-human CD43-FITC monoclonal antibody (BD), anti-human CD45-APC monoclonal antibody (BD), and anti-human CD71-PE monoclonal antibody (BD), and then resuspended in 200 μl FACS buffer and analyzed. For analysis of human HBB, RBCs were first stained with anti-human CD235-APC monoclonal antibody (BD) and then fixed and permeabilized according to the manufacturer’s instructions (BD). Cells were then stained with the anti-human FITC-labeled HB β monoclonal antibody (Santa Cruz Biotech). For sorting of the human CD235a + cells, the peripheral blood cells of mice were collected and stained with anti-human CD235-APC monoclonal antibody (BD) and then sorted according to the instructions. Flow cytometry was performed using FACSAria™ III flow cytometer (BD) and analyzed with FACSDiva™ software (BD).

### Western blot analysis

After sorting, the CD235 + cells were washed twice with cold PBS and then extracted into RIPA lysis buffer with 1 mM PMSF (Beyotime, China) on ice as previously described^38^,^39^. After centrifugation at 15,000 × *g* for 5 min, the protein content was determined using a BCA protein assay kit (Thermo Fisher) according to the manufacturer’s instructions. Equivalent amounts of protein were separated on 15% sodium dodecyl sulfate (SDS)-polyacrylamide gels and blotted onto nitrocellulose membranes. After being blocked at room temperature for 2 h with 5% nonfat milk in TBS with 0.1% Tween-20, the membranes were probed with anti-rabbit human HB beta polyclonal antibody (1:1000; Abgent), anti-GAPDH (1:5000; Cell Signaling Technology), and horseradish peroxidase-conjugated IgG antibodies (1:10000; Cell Signaling Technology). The protein bands were then visualized by exposure to film (Fuji). The volumes of the bands were determined by standard scanning densitometry with normalization of densitometry measures to the expression of GAPDH.

### Statistical analysis

Data are expressed as the mean ± standard error of the mean (SEM) and were compared by one-way analysis of variance (ANOVA). When ANOVA results were significant, differences between groups were assessed with *post-hoc* testing using LSD tests (SPSS version 17.0 for Windows). Differences with *P* < 0.05 were considered statistically significant.

## Additional Information

**How to cite this article**: Ou, Z. *et al*. The Combination of CRISPR/Cas9 and iPSC Technologies in the Gene Therapy of Human β-thalassemia in Mice. *Sci. Rep.* **6**, 32463; doi: 10.1038/srep32463 (2016).

## Figures and Tables

**Table 1:** Summary of routine blood test parameters of six groups of NSI mice before and after transplantation from week 0 to week 4 after irradiation.

	Weight (g)	RBC (×10^12^/l)	HB (g/l)	RE 10^9^/L	RET (%)	IFR (%)
Week 0						
α-MEM group (n = 4)	21.00 ± 0.36	8.65 ± 1.82	143.33 ± 23.18	198.6 ± 79.16	2.31 ± 0.33	38.07 ± 2.40
UCB group (n = 3)	20.17 ± 0.25	7.29 ± 1.06	139.67 ± 10.07	186.07 ± 57.57	2.24 ± 0.55	41.23 ± 0.59
hESC group (n = 4)	21.13 ± 1.90	10.16 ± 1.22	158.25 ± 13.79	276.0 ± 32.44	2.99 ± 0.46	38.70 ± 1.87
piPSC group (n = 5)	20.82 ± 1.00	8.57 ± 1.85	154.0 ± 18.15	253.22 ± 79.93	2.50 ± 0.43	38.46 ± 3.95
ciPSC group (n = 5)	20.16 ± 0.92	8.88 ± 2.48	139.2 ± 10.64	192.66 ± 61.39	2.41 ± 0.35	37.48 ± 5.15
niPSC group (n = 4)	19.93 ± 1.38	10.19 ± 0.44	167.0 ± 11.79	275.73 ± 45.26	3.03 ± 0.45	38.37 ± 3.50
Week 1						
α-MEM group (n = 4)	21.27 ± 0.35	6.36 ± 0.45	108.00 ± 7.21	204.77 ± 72.29	2.74 ± 0.89	35.00 ± 4.79
UCB group (n = 3)	21.07 ± 0.38	7.71. ± 0.67	122.00 ± 9.17	228.00 ± 15.39	3.38 ± 0.30	38.53 ± 3.69
hESC group (n = 4)	21.23 ± 1.32	7.32 ± 0.32	118.25 ±± 7.72	324.13 ± 72.89	4.01 ± 1.07	43.48 ± 2.84
piPSC group (n = 5)	21.12 ± 1.18	7.30 ± 0.92	124.00 ± 13.96	239.10 ± 82.19	2.80 ± 0.87	37.96 ± 11.46
ciPSC group (n = 5)	20.46 ± 1.04	6.67 ± 1.05	114.80 ± 11.26	180.30 ± 53.62	2.61 ± 0.60	33.86 ± 12.88
niPSC group (n = 4)	20.13 ± 1.57	6.84 ± 0.64	114.00 ± 7.55	225.87 ± 60.62	3.32 ± 0.90	34.87 ± 7.74
Week 2						
α-MEM group (n = 4)	21.97 ± 1.17	6.21 ± 1.50	100.33 ± 25.32	390.07 ± 209.36	5.67 ± 1.97	44.37 ± 3.68
UCB group (n = 3)	21.50 ± 0.36	6.64 ± 1.41	126.33 ± 5.03	281.47 ± 72.69	4.21 ± 0.26	45.50 ± 2.36*
hESC group (n = 4)	21.28 ± 0.91	7.01 ± 1.25	122.25 ± 8.54	526.38 ± 82.48	7.68 ± 1.72	39.70 ± 2.73
piPSC group (n = 5)	21.72 ± 0.72	6.89 ± 1.80	97.20 ± 17.09	431.88 ± 256.92	6.30 ± 3.18	36.28 ± 6.27
ciPSC group (n = 5)	21.02 ± 0.69	7.53 ± 1.30	120.00 ± 7.31	278.06 ± 116.70	4.15 ± 0.92	40.64 ± 6.38
niPSC group (n = 4)	20.63 ± 1.36	6.50 ± 0.31	120.00 ± 8.54	278.06 ± 116.70	8.85 ± 2.72	38.63 ± 2.39
Week 3						
α-MEM group (n = 4)	22.30 ± 1.06	6.22 ± 1.97	114.67 ± 14.50	246.00 ± 60.91	3.81 ± 0.55	46.03 ± 2.14
UCB group (n = 3)	22.0 ± 0.44	8.36 ± 0.53	133.00 ± 9.85	292.20 ± 34.66	3.17 ± 0.86	39.30 ± 9.36
hESC group (n = 4)	22.08 ± 0.97	7.67 ± 0.48	115.75 ± 11.30	227.65 ± 103.95	3.80 ± 0.74	44.83 ± 3.42
piPSC group (n = 5)	22.28 ± 1.17	6.44 ± 1.08	116.80 ± 12.36	201.74 ± 63.14	2.84 ± 0.77	43.62 ± 4.37
ciPSC group (n = 5)	21.34 ± 0.60	7.12 ± 0.90	107.80 ± 27.06	249.06 ± 97.20	3.53 ± 0.42	48.52 ± 3.18
niPSC group (n = 4)	21.37 ± 0.83	6.06 ± 0.81	117.00 ± 11.14	191.93 ± 68.77	3.13 ± 081	40.67 ± 8.18
Week 4						
α-MEM group (n = 4)	22.83 ± 0.81	7.63 ± 0.57	121.33 ± 2.52	295.10 ± 125.56	4.08 ± 1.36	42.57 ± 1.97
UCB group (n = 3)	22.50 ± 0.36	7.62 ± 0.28	124.00 ± 3.46	243.50 ± 8.06	3.20 ± 0.17	40.90 ± 7.12
hESC group (n = 4)	22.60 ± 1.10	6.84 ± 1.07	130.00 ± 10.61	190.98 ± 60.02	3.10 ± 0.48	38.13 ± 5.06
piPSC group (n = 5)	22.48 ± 1.49	8.11 ± 1.03	120.00 ± 14.85	258.44 ± 87.58	3.37 ± 1.13	44.02 ± 4.32
ciPSC group (n = 5)	21.90 ± 0.70	7.66 ± 0.45	127.80 ± 8.04	237.96 ± 24.81	3.10 ± 0.42	45.06 ± 8.92
niPSC group (n = 4)	21.93 ± 0.95	6.57 ± 0.80	115.67 ± 11.71	177.13 ± 29.68	3.02 ± 0.58	41.20 ± 4.64
	**MCV (fl)**	**MCH (pg)**	**MCHC (g/L)**	**RDW (%)**	**MFR (%)**	
Week 0						
α-MEM group (n = 4)	61.93 ± 3.66	16.0 ± 0.10	271.00 ± 13.00	16.40 ± 1.28	16.73 ± 3.55	
UCB group (n = 3)	61.63 ± 1.25	16.0 ± 0.20	265.00 ± 11.79	14.73 ± 0.60	16.67 ± 1.86	
hESC group (n = 4)	57.40 ± 1.19	16.05 ± 0.25	280.00 ± 8.76	15.85 ± 1.06	15.55 ± 1.40	
piPSC group (n = 5)	60.58 ± 1.78	16.20 ± 0.66	262.40 ± 13.78	16.90 ± 2.07	16.38 ± 1.31	
ciPSC group (n = 5)	59.58 ± 2.50	16.72 ± 1.21	282.00 ± 25.61	17.76 ± 1.72	16.76 ± 1.94	
niPSC group (n = 4)	57.57 ± 4.50	16.40 ± 0.17	286.00 ± 20.42	17.00 ± 1.11	14.87 ± 1.57	
Week 1						
α-MEM group (n = 4)	60.10 ± 0.17	16.70 ± 0.56	278.00 ± 9.00	16.73 ± 1.81	19.36 ± 4.03	
UCB group (n = 3)	58.20 ± 3.54	15.87 ± 0.42	272.67 ± 13.65	15.17 ± 1.16	16.30 ± 1.93	
hESC group (n = 4)	35.13 ± 1.90	16.15 ± 0.54	282.50 ± 4.51	17.13 ± 1.76	15.33 ± 1.82	
piPSC group (n = 5)	56.42 ± 1.28	16.02 ± 0.23	284.20 ± 9.44	14.88 ± 1.19	17.60 ± 1.73	
ciPSC group (n = 5)	58.88 ± 5.33	16.76 ± 1.55	292.60 ± 27.40	15.98 ± 2.31	17.06 ± 3.41	
niPSC group (n = 4)	57.60 ± 1.18	16.70 ± 0.50	290.33 ± 8.33	17.00 ± 1.73	19.27 ± 2.22	
Week 2						
α-MEM group (n = 4)	57.87 ± 0.75	16.57 ± 0.12	295.33 ± 9.29	17.87 ± 3.25	18.50 ± 2.76	
UCB group (n = 3)	57.70 ± 0.72	16.07 ± 0.49	282.33 ± 3.51	15.90 ± 0.60	18.40 ± 1.10	
hESC group (n = 4)	58.20 ± 2.01	16.33 ± 0.28	279.50 ± 11.81	19.95 ± 1.82	16.28 ± 0.94	
piPSC group (n = 5)	58.90 ± 1.30	16.78 ± 0.93	284.80 ± 9.36	18.86 ± 3.70	15.38 ± 4.20	
ciPSC group (n = 5)	60.44 ± 1.68	16.60 ± 0.76	278.00 ± 13.78	19.32 ± 2.55	17.36 ± 1.96	
niPSC group (n = 4)	60.70 ± 3.01	16.97 ± 0.55	280.00 ± 9.17	21.90 ± 2.00	15.20 ± 0.36	
Week 3						
α-MEM group (n = 4)	60.23 ± 0.85	16.93 ± 0.55	280.67 ± 10.07	17.87 ± 0.42	17.60 ± 0.44	
UCB group (n = 3)	57.43 ± 1.07	16.57 ± 1.17	277.33 ± 8.50	15.87 ± 1.26	17.57 ± 2.02	
hESC group (n = 4)	58.25 ± 2.51	17.10 ± 0.46	298.00 ± 3.92	20.08 ± 1.04	17.80 ± 0.42	
piPSC group (n = 5)	59.42 ± 4.46	16.48 ± 0.51	278.40 ± 20.55	17.54 ± 0.12	17.46 ± 2.37	
ciPSC group (n = 5)	60.44 ± 3.63	16.60 ± 0.93	275.00 ± 16.22	18.30 ± 2.10	18.14 ± 1.15	
niPSC group (n = 4)	59.90 ± 2.19	17.23 ± 0.91	294.33 ± 20.50	19.47 ± 0.21	18.70 ± 3.62	
Week 4						
α-MEM group (n = 4)	60.03 ± 3.70	16.80 ± 0.81	270.33 ± 26.69	16.73 ± 2.35	17.07 ± 1.17	
UCB group (n = 3)	57.57 ± 1.18	16.30 ± 0.46	283.00 ± 9.54	16.07 ± 1.10	19.36 ± 3.49	
hESC group (n = 4)	57.63 ± 1.90	17.05 ± 0.62	292.25 ± 6.45	17.65 ± 0.98	18.80 ± 2.54	
piPSC group (n = 5)	59.02 ± 3.71	16.70 ± 0.72	279.80 ± 11.92	16.90 ± 1.01	17.30 ± 1.16	
ciPSC group (n = 5)	62.06 ± 2.20	17.20 ± 1.17	272.20 ± 14.92	17.72 ± 1.14	18.84 ± 0.84	
niPSC group (n = 4)	63.00 ± 5.99	17.27 ± 1.00	286.00 ± 17.78	20.03 ± 1.79	16.60 ± 1.04	

Values represent mean ± SD. Statistical significance was determined for HB of the α-MEM, niPSC, UCB, and hESC groups compared with that of piPSC group. RBC indicates red blood cell count; HB, hemoglobin; RET, reticulocyte count; IFR, immature reticulocyte fraction; MCV, mean corpuscular volume; MCH, mean corpuscular hemoglobin; MCHC, mean corpuscular hemoglobin concentration; RDW, red cell distribution width; MFR, middle fluorescent reticulocyte.
